# Feline XRCC4 undergoes rapid Ku‐dependent recruitment to DNA damage sites

**DOI:** 10.1002/2211-5463.13363

**Published:** 2022-02-18

**Authors:** Manabu Koike, Yasutomo Yutoku, Aki Koike

**Affiliations:** ^1^ Institute for Quantum Medical Science National Institutes for Quantum Science and Technology Chiba Japan; ^2^ Department of Regulatory Biology Faculty of Science Saitama University Saitama Japan

**Keywords:** cat, companion animal, feline, Ku80, XRCC4

## Abstract

Radiation and chemotherapy resistance remain some of the greatest challenges in human and veterinary cancer therapies. XRCC4, an essential molecule for nonhomologous end joining repair, is a promising target for radiosensitizers. Genetic variants and mutations of *XRCC4* contribute to cancer susceptibility, and *XRCC4* is also the causative gene of microcephalic primordial dwarfism (MPD) in humans. The development of clinically effective molecular‐targeted drugs requires accurate understanding of the functions and regulatory mechanisms of XRCC4. In this study, we cloned and sequenced the cDNA of feline *XRCC4*. Comparative analysis indicated that sequences and post‐translational modification sites that are predicted to be involved in regulating the localization of human XRCC4, including the nuclear localization signal, are mostly conserved in feline XRCC4. All examined target amino acids responsible for human MPD are completely conserved in feline XRCC4. Furthermore, we found that the localization of feline XRCC4 dynamically changes during the cell cycle. Soon after irradiation, feline XRCC4 accumulated at laser‐induced DNA double‐strand break (DSB) sites in both the interphase and mitotic phase, and this accumulation was dependent on the presence of Ku. Additionally, XRCC4 superfamily proteins XLF and PAXX accumulated at the DSB sites. Collectively, these findings suggest that mechanisms regulating the spatiotemporal localization of XRCC4 are crucial for XRCC4 function in humans and cats. Our findings contribute to elucidating the functions of XRCC4 and the role of abnormal XRCC4 in diseases, including cancers and MPD, and may help in developing XRCC4‐targeted drugs, such as radiosensitizers, for humans and cats.

## Graphical abstract

In this study, feline XRCC4 cDNA was cloned and analyzed. Our data suggest that the mechanisms controlling the spatiotemporal localization of XRCC4 are critical for XRCC4 function. Our findings contribute to elucidating the XRCC4 functions and the role of abnormal XRCC4 in diseases, including cancers and microcephalic primordial dwarfism, and may help in developing XRCC4‐targeted drugs for humans and cats.

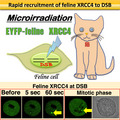

**Abbreviations**
CLSM: confocal laser‐scanning microscope
DIC: differential interference contrast
DSB: DNA double‐strand break
MPD: microcephalic primordial dwarfism
NHEJ: nonhomologous end joining
NLS: nuclear localization signal
PTMs: post‐translational modifications
SIM: SUMO interaction motif
SUMO: small ubiquitin‐like modifier

Pet animals, such as cats and dogs, play the role of companion animals and are becoming increasingly important in the human society. Cancer is a leading cause of death of these pets, and innovations in cancer treatment are desired [1, 2]. However, as well as in human cancer therapies, resistance against chemo‐ and radiotherapy remains among the greatest challenges in veterinary cancer therapy. In humans, advanced radiotherapies, such as heavy ion radiation therapy, and advanced chemotherapies, such as molecular targeting therapies including synthetic lethal approaches, have resulted in successful outcomes in cancer, but sensitizer development is indispensable for treating intractable cancer [3, 4]. As conventional radiotherapy is becoming widely used for cancer treatment in dogs and cats, advanced treatments will be employed in the near future [3, 5, 6]. Clinical studies have shown that the effects of radiation therapy differ between cats and dogs (in terms of tumor responses and normal tissue toxicity) [5]. Spontaneous cancers in companion animals represent excellent models for cancer research in both veterinary and human medicine [1, 6]. However, no studies have been conducted on advanced treatments in cats, namely, a synthetic lethal approach targeting DNA repair mechanisms.

DNA double‐strand break (DSB) is the most cytotoxic DNA damage and is predominantly repaired via the nonhomologous end joining (NHEJ) pathway [3, 7]. The NHEJ pathway is an attractive target for strategies aimed at increasing the susceptibility of tumors to anticancer treatments, considering the fact that DSB cytotoxicity is the mechanism of action of numerous anticancer therapies such as conventional radiotherapies and chemotherapies [3, 8, 9]. As cancer cells often characterized by abnormalities in the DNA repair mechanism, drug development based on synthetic lethality aimed at other remaining DNA repair mechanisms, for example, poly (ADP‐ribose) polymerase inhibitors for treating *BRCA1/2*‐mutated ovarian cancers, is clinically prominent [4, 8, 9]. The DSB sensor Ku (Ku70 and Ku80 heterodimer), XRCC4, and DNA ligase IV are essential factors for DSB repair by the NHEJ pathway [7, 10, 11]. The binding of XRCC4 with DNA Ligase IV stabilizes DNA Ligase IV and enhances its rejoining activity, which is essential for the final ligation of DSB through NHEJ. This step is believed to be assisted by XLF (also called NHEJ1 or Cernunnos). Meanwhile, XRCC4 binds to XLF to form filaments that bridge broken DNA during NHEJ, independent of DNA ligase IV activity [12]. This DNA bridging by XRCC4/XLF filaments appears to be regulated by the phosphorylation of C‐terminal tails of both proteins [13]. In addition, PAXX (also called XLS or C9orf142), which is structurally classified into the XRCC4 superfamily along with XRCC4 and XLF, has been suggested to play a role in human NHEJ [14, 15, 16]. On the other hand, several studies have demonstrated that lack of XLF in mice can be compensated with ATM, 53BP1, DNA‐PKcs, H2AX, MRI/Cyren, Rag2, and PAXX [17, 18, 19, 20, 21]. Interestingly, mice lacking XLF or PAXX were viable and did not have an overt phenotype, whereas mice lacking XRCC4 were late embryonic lethal [17, 18, 19, 20, 22]. Meanwhile, XLF/PAXX double knockout mice died during late embryonic development, demonstrated significant accumulation of DSBs and neural apoptosis, like the XRCC4 single knockout mice [19]. These findings strongly suggest that the functions of XRCC4 and XLF are not completely identical in NHEJ, in spite of the fact that these two proteins have similar protein structure and roles.

XRCC4 silencing increases the radiosensitivity of various cancer cells in humans, including breast, colon, and lung cancers [23, 24, 25]. Further, genetic variants such as the single nucleotide polymorphism p.Ala247Ser, and other XRCC4 mutations, contribute to cancer susceptibility in non‐*BRCA1/2* breast cancer, oral cancer, and hepatocellular carcinoma in humans [26, 27, 28]. *XRCC4* is also the causative gene of human microcephalic primordial dwarfism (MPD), and various *XRCC4* mutations have been observed in MPD patients without any overt immunodeficiency [29, 30]. In order to develop efficacious molecular‐targeted drugs, it is essential to elucidate the functions and regulatory mechanisms of targeted proteins in the organism to be treated. However, there are no reports concerning feline XRCC4 proteins and NHEJ repair as promising radiosensitizer targets, except for our report regarding the mechanisms underlying accumulation of feline XLF at DSB sites [31].

NHEJ, which rapidly progresses immediately after DNA damage, appears to be finely controlled by protein interactions and post‐translational modifications (PTMs) of DNA repair proteins. Here, to obtain the fundamental information that would enable us to comparatively analyze the XRCC4 regulatory mechanism, we cloned and sequenced feline XRCC4 cDNA and studied the expression, localization, and recruitment of XRCC4 proteins at the DSB in feline cells. Additionally, we performed an interspecies comparative analysis to understand the mechanisms regulating XRCC4 functions.

## Materials and methods

### Cell lines and cell cultures

Crandell‐Rees feline kidney [CRFK, Health Science Research Resources BANK (HSRRB), Osaka, Japan], normal human diploid lung fibroblast (TIG‐1, HSRRB), mouse embryonic fibroblast (NIH3T3; Riken Cell Bank, Tsukuba, Japan), Chinese hamster ovary (CHO‐K1; Riken Cell Bank), Ku80‐deficient CHO‐K1 mutant (xrs‐6), XRCC4‐deficient CHO‐K1 mutant (XR‐1), and human colon cancer cell lines (HCT116; Riken Cell Bank) were cultured as previously described [31, 32, 33, 34, 35]. A XRCC4‐deficient cell line HCT116 (XRCC4^−/−^; Riken Cell Bank) was cultured in Dulbecco's modified Eagle's medium supplemented with antibiotics and 10% fetal bovine serum and antibiotics and maintained in a humidified incubator at 37 °C under an atmosphere of 5% CO_2_.

### Western blot analysis

Preparation of total cell lysate and western blot analysis were carried out as previously described [31, 36, 37] with the following modifications: The total proteins were electrophoresed on Extra PAGE One Precast Gel 5–20% (Nacalai Tesque, Kyoto, Japan, 13064‐64); the molecular weight marker used was a 3‐Color prestained XL‐ladder (APRO Science, Tokushima, Japan, SP‐2140); and the membranes were blocked in Blocking One (Nacalai Tesque, 03953‐95) or ECL Prime Blocking reagent (GE Healthcare Bio‐Sciences. Corp., Piscataway, NJ, USA, RPN418) for 30 min at room temperature. The following antibodies were used: goat anti‐XRCC4 polyclonal antibody (C‐20; Santa Cruz Biotechnology, Santa Cruz, TX, USA, sc‐8285), anti‐C9orf142 (PAXX) polyclonal antibody (Abcam, Cambridge, UK, ab126353), rabbit anti‐GFP polyclonal antibody (FL; Santa Cruz Biotechnology, sc‐8334), and mouse anti‐β‐actin monoclonal antibody (AC‐15; Sigma‐Aldrich, St. Louis, MO, USA, A5441). In accordance with the manufacturer's instructions, protein bands were detected using a Select Western Blotting Detection System (GE Healthcare Bio‐Sciences. Corp., RPN2235) or Chemi‐Lumi One Ultra (Nacalai Tesque, 11644‐40), and visualized using the ChemiDoc XRS System (Bio‐Rad, Hercules, CA, USA).

### Feline XRCC4 cloning and expression vectors

Cloning of feline *XRCC4* cDNA was performed as previously described [31, 35] with the following modifications: Oligonucleotide primers used to amplify feline *XRCC4* cDNA from a cat (male) cDNA library (Zyagen, San Diego, CA, USA, FD‐401) were designed based on the predicted XRCC4 genomic sequence of a female cat (*Felis catus*; XM_011284381.1 and XM_011284386.1). *EcoR1* and *BamH1* restriction enzyme sites were incorporated on the 5′ end of the sense (Feline XRCC4 F) and antisense primers (Feline XRCC4 R), respectively. PCR amplification with sense (Feline XRCC4 F: 5′‐GAATTCTATGGAAAGAAAAGTAAGCAGAA‐3′) and antisense (Feline XRCC4 R: 5′‐GGATCCTTAAATCTCATCAAAGAGGTCTTCTG‐3′) primers was performed on a PCR Thermal Cycler Dice (Takara Bio Inc., Shiga, Japan) using LA Taq polymerase (Takara Bio Inc., PR002A). Predenaturation was carried out for 5 min at 94 °C followed by 35 PCR amplification cycles. Each cycle consisted of a denaturation step at 94 °C for 1 min, annealing at 56 °C for 1 min and extension at 72 °C for 1 min, followed by a final 4‐min extension step at 72 °C. PCR products were subcloned into the pCR4‐TOPO TA vector (Invitrogen, Carlsbad, CA, USA, K457501) (pCR4‐feline *XRCC4* plasmid), and the nucleotide sequences were sequenced using sequencing primers T3 and T7. *XRCC4* cDNA from pCR4‐feline *XRCC4* plasmid was subcloned into the *Eco*RI and *Bam*HI sites of pEYFP‐C1 (pEYFP‐feline *XRCC4*), and the inserts were validated by sequencing. Other PCR primers used in this study were as follows: Feline XRCC4 F1: 5′‐GCGAATGAGAGGTGACCAGAAATG‐3′, Feline XRCC4 R1: 5′‐CTTGGTTTTCCGCAATGGTGTCC‐3′, Feline XRCC4 F2: 5′‐TGAAGAAAGTGAGAACCTGCCTGATCC‐3′, and Feline XRCC4 R2: 5′‐GCAGCCATGTCCTTTTGAGACAGTTA‐3′. These PCR primers were used to validate the synthetic primer sequence (Feline XRCC4 F and Feline XRCC4 R). We confirmed that the sequence around ATG is based on the cognate sequence.

### Plasmids and DNA transfections

pEYFP‐feline *XRCC4,* pEYFP‐feline *XLF,* or pEYFP‐C1 were transfected into cells using Lipofectamine 3000 (Invitrogen, L3000‐008) [31]. Cells were cultured for 2 days after transfection, and then, cell images were acquired using a FV300 confocal laser‐scanning microscope (CLSM) system (Olympus, Tokyo, Japan), as described previously [38, 39].

### DNA damage induction using micro‐laser and cell imaging

Local DNA damage induction using micro‐laser and subsequent cell imaging was performed as previously described [39, 40]. Briefly, DSBs were locally induced using a 405‐nanometer diode laser equipped by an FV300 CLSM system (Olympus). Images of live and/or fixed cells expressing EYFP‐feline XRCC4, EYFP‐feline XLF, or EYFP alone were obtained using an FV300 CLSM system (Olympus). Immunocytochemistry was performed previously described [31, 39] using the following antibodies: rabbit anti‐Ku80 polyclonal (H300; Santa Cruz Biotechnology, sc‐9034), rabbit anti‐Ku70 polyclonal (H308; Santa Cruz Biotechnology, sc‐9033), mouse anti‐γH2AX monoclonal (JBW301; Upstate Biotechnology Inc., Lake Placid, NY, USA, 05‐636), rabbit anti‐γH2AX polyclonal (Cell Signaling Technology, Beverly, MA, USA, 2577S), rabbit anti‐C9orf142 (PAXX) polyclonal (Abcam, ab126353), and Alexa Fluor 568‐conjugated secondary [Molecular Probes, Eugene, OR, USA, A11036 (anti‐rabbit); A‐11031 (anti‐mouse)].

### XRCC4 sequence comparisons among mammalian orthologs

The amino acid sequence of feline XRCC4 was compared with sequences of canine, human, chimpanzee, and mouse XRCC4 using the Pairwise Sequence Alignment EMBOSS Needle [The European Bioinformatics Institute (EMBL‐EBI); https://www.ebi.ac.uk/Tools/psa/] [41].

## Results

### Molecular cloning and sequence alignment of feline XRCC4

**Fig. 1:**
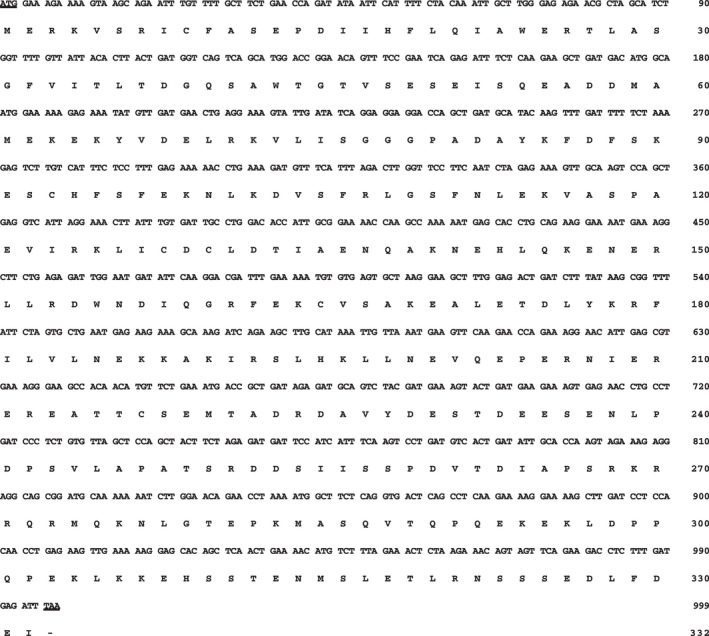
Nucleotide and deduced amino acid sequences of feline *XRCC4* cDNA (*Felis catus*, GenBank accession number: LC309245). The CDS of feline *XRCC4* is composed of 999 bp encoding 332 amino acids residues. Numbers on the right refer to nucleotides (top) and amino acids (bottom). The start (ATG) and stop (TAA) codons are underlined.

**Fig. 2:**
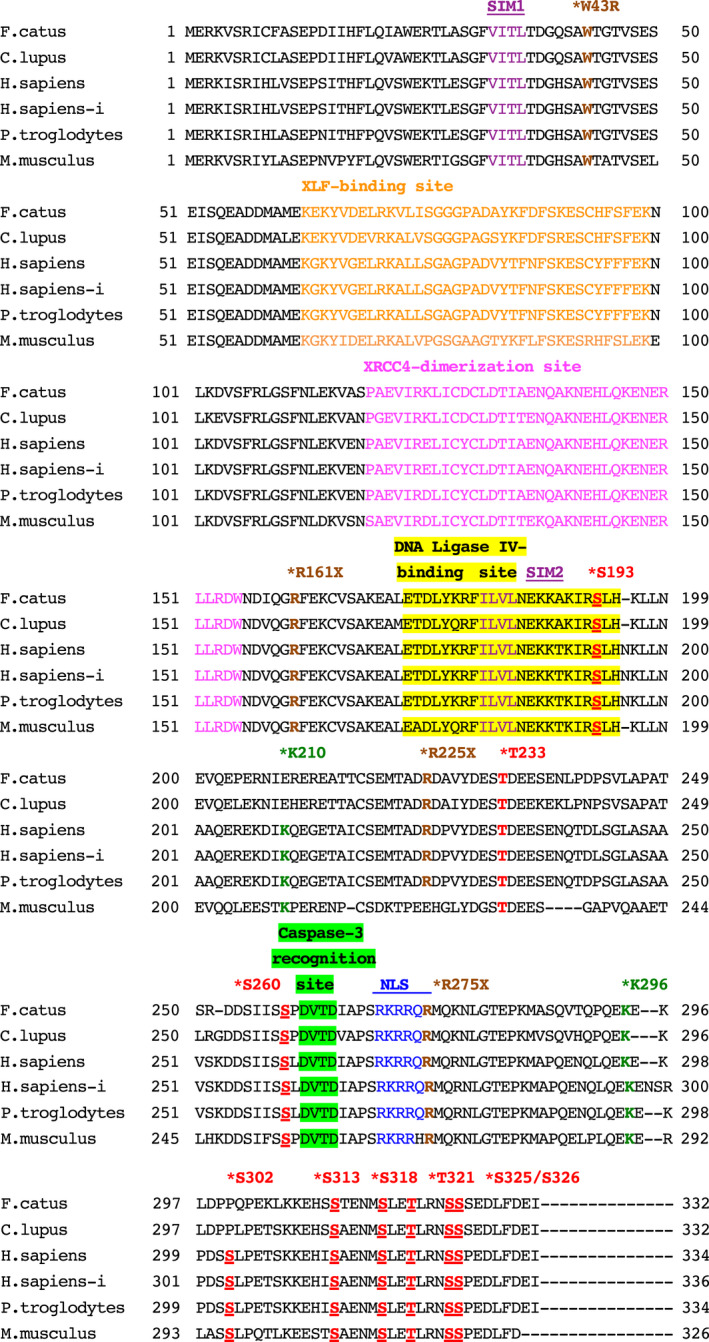
XRCC4 sequence alignment. Amino acid sequences of XRCC4 from cat (*Felis catus*, GenBank accession number: LC309245), dog (*Canis lupus familiaris*, GenBank accession number: LC168634), human (*Homo sapiens*, GenBank accession number: AAC50339.1), human isoform (H.sapiens‐i; *H. sapiens*, GenBank accession number: NP_071801.1), chimpanzee (*Pan troglodytes*, GenBank accession number: NP_001267327.1), and mouse (*Mus musculus*, GenBank accession number: NP_082288.1) species. The XLF‐binding site [amino acids (aa) 63–99, highlighted in orange], the region of XRCC4‐dimerization (aa 119–155, highlighted in pink), and DNA ligase IV‐binding site (aa 173–195, highlighted in yellow) of XRCC4 are indicated [18]. The location of a caspase‐3 recognition site (_262_DVTD_265_) in human XRCC4 and a putative NLS sequence (_270_RKRRQR_275_) in human XRCC4 [42, 45] is shown. The location of certain disease‐associated mutations in human MPD patients (W43R, R161X, R225X, and R275X), of SUMO modification site (K210), polyubiquitylation site (K296), CK2 phosphorylation site (T233), and major DNA‐PK phosphorylation sites (S260 and S318) in the human sequences (AAC50339.1) [7, 29, 30, 43, 46] is marked with asterisks. The location of SIM‐1 and SIM‐2 is indicated [44]. The eight phosphorylation site locations (S193, S260, S302, S313, S318, T321, S325, S326), which impact the stability and DNA bridging capacity of XRCC4/XLF complexes in the human XRCC4 sequence [13], are marked with asterisks and underlined.

First, feline *XRCC4* cDNA was cloned using a cat testis library as template and then sequenced. We isolated a 999‐nucleotide open reading frame encoding the feline XRCC4 protein (332 amino acids) and deposited in the DDBJ/EMBL/NCBI database (accession number: LC309245; Fig. 1). Comparative sequence analysis of XRCC4 orthologs from different species revealed that feline XRCC4 has 81.1% (85.9%), 81.4% (86.2%), 88.9% (97.0%), and 72.6% (79.8%) amino acid identity (similarity) with the corresponding human, chimpanzee, dog, and mouse orthologs, respectively (Table 1). Alternatively, mouse XRCC4 exhibited 74.0% (81.4%), 74.6% (81.4%), and 73.3% (81.4%) amino acid identity (similarity) with the corresponding human, chimpanzee, and dog orthologs, respectively. Protein interactions, protein cleavage, and PTMs, including phosphorylation, SUMOylation, and ubiquitylation of XRCC4, might play a key role in regulating the functions of XRCC4 in human cells [7, 42, 43, 44]. To examine whether these modification sites and functional domains are evolutionally conserved in feline XRCC4, the amino acid sequence of feline XRCC4 was compared with that of other animal species (Fig. 2). There are an XLF‐binding site [amino acids (aa) 63–99], a region of XRCC4‐dimerization [(aa) 119–155], and a DNA ligase IV‐binding site [(aa) 173–195] in human XRCC4 [18]. The amino acid sequences of these sites involved in protein interactions are evolutionarily conserved among cats, dogs, humans, chimpanzees, and mice. We also identified that the caspase‐3 recognition site (_262_DVTD_265_)—used in apoptosis‐dependent cleavage—and the putative nuclear localization signal (NLS) sequence (_270_RKRRQR_275_) present in human XRCC4 [42, 45] are perfectly conserved in feline XRCC4. Recently, it has been reported that human XRCC4 is modified with the small ubiquitin‐like modifier (SUMO)2 [44]. Two SUMO interaction motifs, SIM‐1 and SIM‐2, are presented in human XRCC4. Additionally, it has been reported that human XRCC4 (K210) is SUMOylated, and this SUMOylation is essential for the nuclear localization of XRCC4 [46]. We found that SIM‐1 and SIM‐2 are perfectly conserved among cats, dogs, humans, chimpanzees, and mice. However, our data revealed that the amino acid K210 in human XRCC4 is not conserved in feline or canine XRCC4 [35]. Furthermore, we found that the polyubiquitylation site (K296), CK2 phosphorylation site (T233), and DNA‐PK major phosphorylation sites (S260 and S318) in human XRCC4 [7, 43] are evolutionarily conserved in the corresponding orthologs in cats and other examined species. Moreover, the eight phosphorylation sites (S193, S260, S302, S313, S318, T321, S325, S326), which control the stability and DNA bridging ability of XRCC4/XLF complexes in human XRCC4, are mostly conserved in the XRCC4 of all examined species [13]. Certain XRCC4 mutations induce the development of the human disease MPD [29, 30]. We found that all of the examined target amino acids (W43, R161, R225, and R275) are perfectly conserved in feline XRCC4, whereas R225 is not conserved in murine XRCC4.

**Table 1:** Comparisons of XRCC4 sequence with the sequences of orthologs.

Protein (accession number^a^)	Identity withfeline XRCC4^b^	Similarity tofeline XRCC4	Identity withmouse XRCC4	Similarity tomouse XRCC4
Human XRCC4 (AAC50339.1)	81.1%	85.9%	74.0%	81.4%
Chimpanzee XRCC4 (NP_001267327.1)	81.4%	86.2%	74.6%	81.4%
Canine XRCC4 (LC168634)	88.9%	97.0%	73.3%	81.4%
Mouse XRCC4 (NP_082288.1)	72.6%	79.8%	–	–

^a^ Accession number in DDBJ/EMBL/NCBI database.

^b^ Feline XRCC4 (LC309245).

### XRCC4 expression in feline cells

**Fig. 3:**
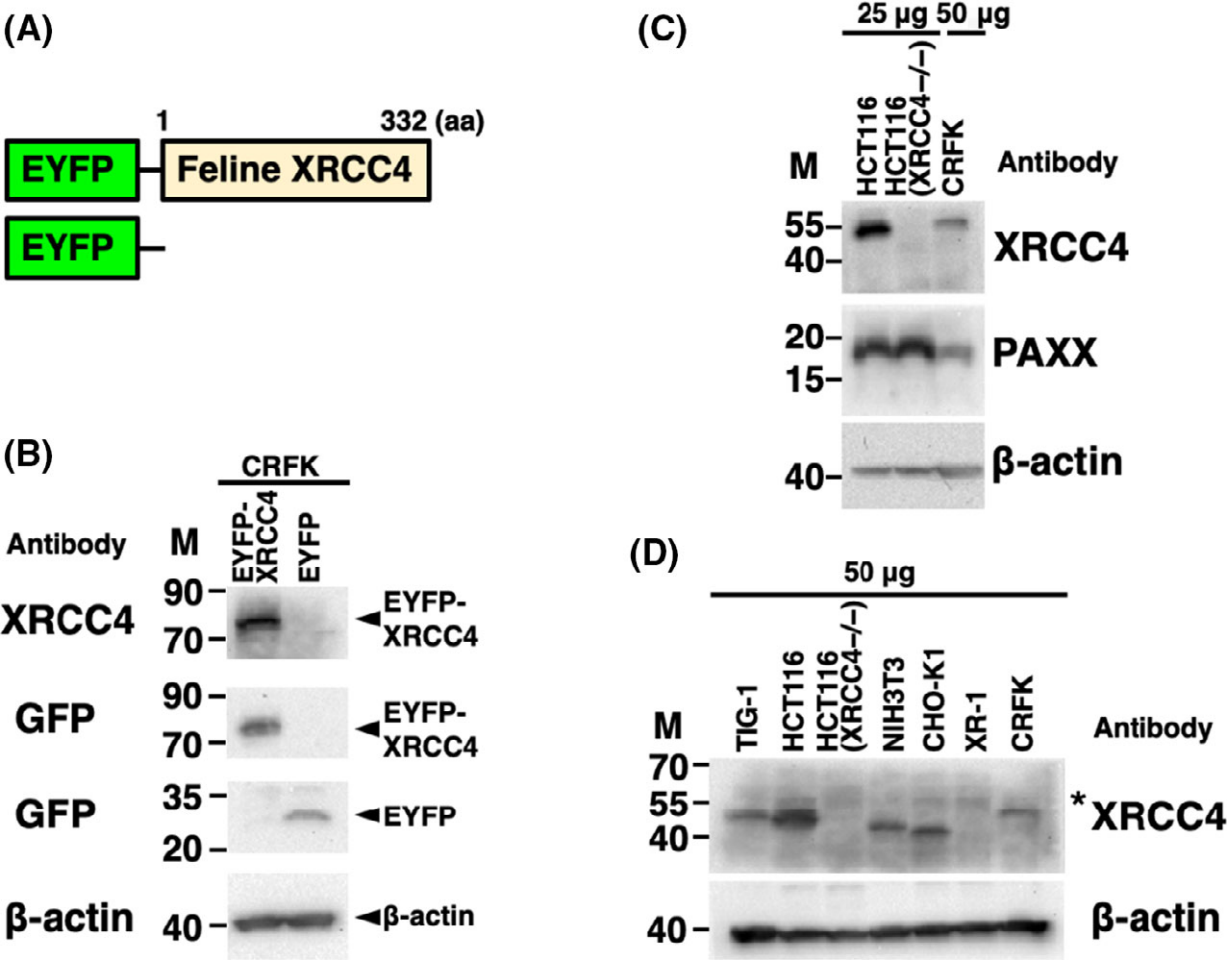
XRCC4 expression in feline cells. (A) Scheme relative to the EYFP‐feline XRCC4 chimeric protein (EYFP‐feline XRCC4) and control protein (EYFP). (B) EYFP‐feline XRCC4 expression in CRFK cells. Extracts from cells transiently expressing EYFP‐feline XRCC4 or EYFP were analyzed by western blotting using anti‐XRCC4, anti‐GFP, and anti‐β‐actin antibodies. (C) XRCC4 and PAXX expression in feline and human cells. Total cell proteins from the feline (CRFK, 50 μg per lane) and human cell lines (HCT116 and HCT116 (XRCC4^−/−^), 25 μg per lane) were analyzed by western blotting using anti‐XRCC4, anti‐PAXX, or anti‐β‐actin antibodies. (D) XRCC4 expression in feline and other mammalian cells. Total cell lysates from the feline (CRFK), three human [TIG‐1, HCT116, and HCT116(XRCC4^−/−^)], one mouse (NIH3T3), and two hamster cell lines (CHO‐K1 and XR‐1) were analyzed by western blotting using anti‐XRCC4 or anti‐β‐actin antibodies. *: nonspecific band; M, molecular weight marker (kDa).

To generate cells transiently expressing EYFP‐feline XRCC4, the expression vector pEYFP‐C1 containing feline *XRCC4* (pEYFP‐feline *XRCC4*) or pEYFP‐C1 was transfected into CRFK cells (Fig. 3A). Western blotting using anti‐XRCC4 and anti‐GFP antibodies showed that that the fusion protein EYFP‐XRCC4 or EYFP was expressed in the transfectant cell lines (Fig. 3B). In addition, these results indicated that the antibody used can detect feline XRCC4. Previously, we have shown that in addition to feline Ku70 and Ku80, the NHEJ factor XLF (a member of the XRCC4 superfamily) is expressed in CRFK cells [31]. First, we verified the expression of two other NHEJ factors (XRCC4 superfamily members) in CRFK and human [HCT116 and HCT116 (XRCC4^−/−^)] cell lines, by western blotting. As shown in Fig. 3C, the expression of feline XRCC4 and PAXX was detected in CRFK cells. Expectedly, human PAXX, but not human XRCC4, was detected in HCT116 (XRCC4^−/−^) cells, whereas both human PAXX and XRCC4 were detected in HCT116 cells. Next, we examined XRCC4 expression in CRFK cells, three human cell lines [TIG‐1, HCT116, and HCT116 (XRCC4^−/−^)], the mouse cell line (NIH3T3), and two hamster cell lines (CHO‐K1 and XR‐1). XRCC4 expression was detected in feline, human, mouse, and hamster XRCC4, whereas it was not detected in the extracts of two XRCC4‐deficient cell lines, XR‐1 and HCT116 (XRCC4^−/−^) (Fig. 3D). Interestingly, the electrophoretic mobility of feline XRCC4 differed from that of human, mouse, and hamster XRCC4. These results support the possibility that a part of PTMs on feline XRCC4 may be different from those in human, mouse, and hamster XRCC4.

### Subcellular localization of feline XRCC4 during the cell cycle

**Fig. 4:**
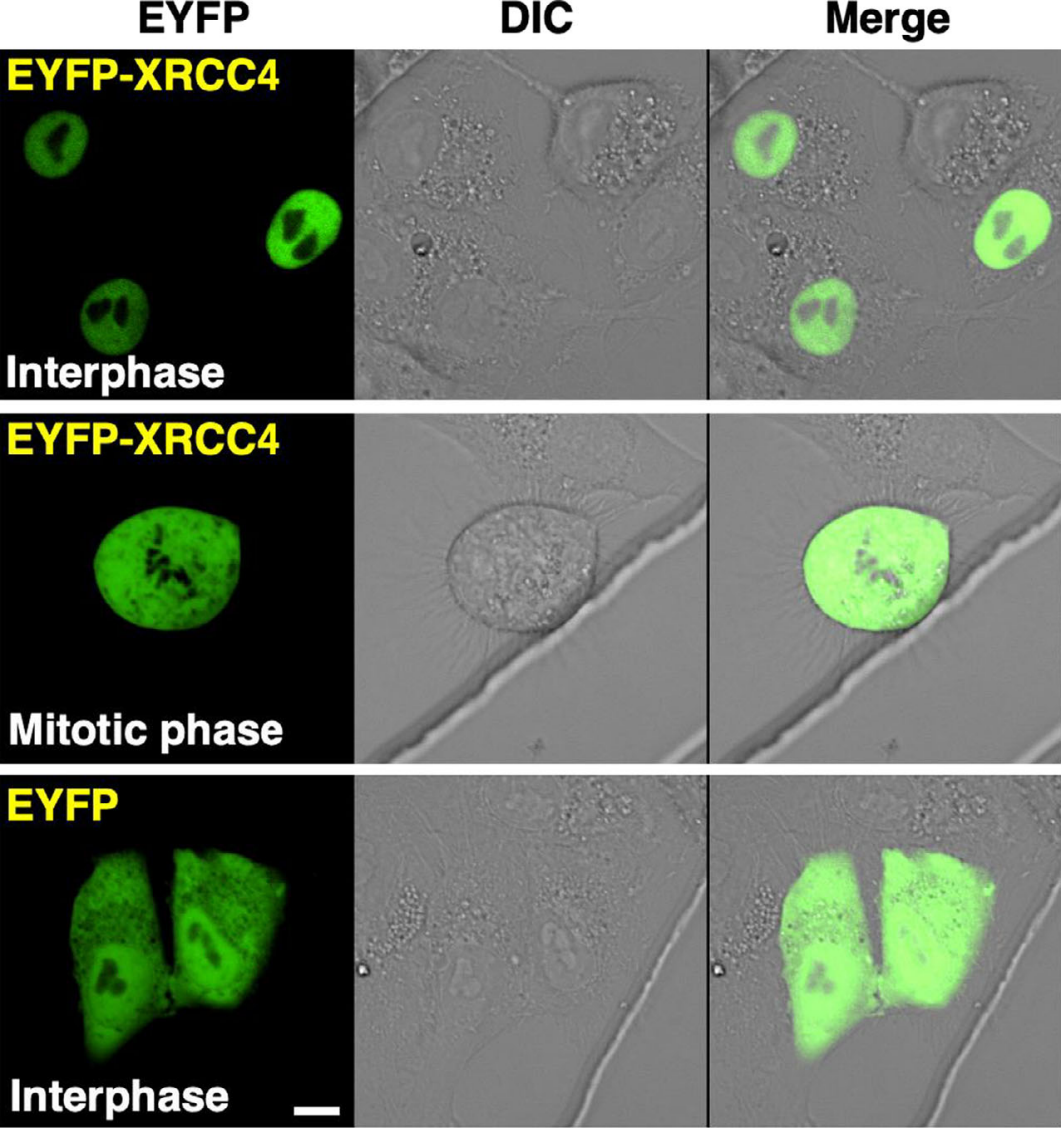
Feline XRCC4 subcellular localization. Cells transiently expressing EYFP‐feline XRCC4 or EYFP were examined by confocal laser microscopy. EYFP images for the same cells are shown alone (left panel) or merged (right panel) with the corresponding differential interference contrast (DIC; middle panel) images. ‘Interphase’ and ‘Mitotic phase’ indicate cells in the interphase and mitotic phase, respectively. Bar, 10 μm.

Nest, we observed CRFK cells transiently expressing EYFP‐feline XRCC4 to examine XRCC4 localization in live feline cells. Confocal laser microscopy revealed that EYFP‐feline XRCC4 localizes in the nucleoplasm in interphase cells, whereas it localizes throughout the mitotic cell (except at the mitotic chromosome) (Fig. 4). As expected, the control (EYFP) was localized throughout the cell (except in the nucleolus) in pEYFP‐transfected cells.

### Ku‐dependent recruitment of feline XRCC4 to the sites of DSB

**Fig. 5:**
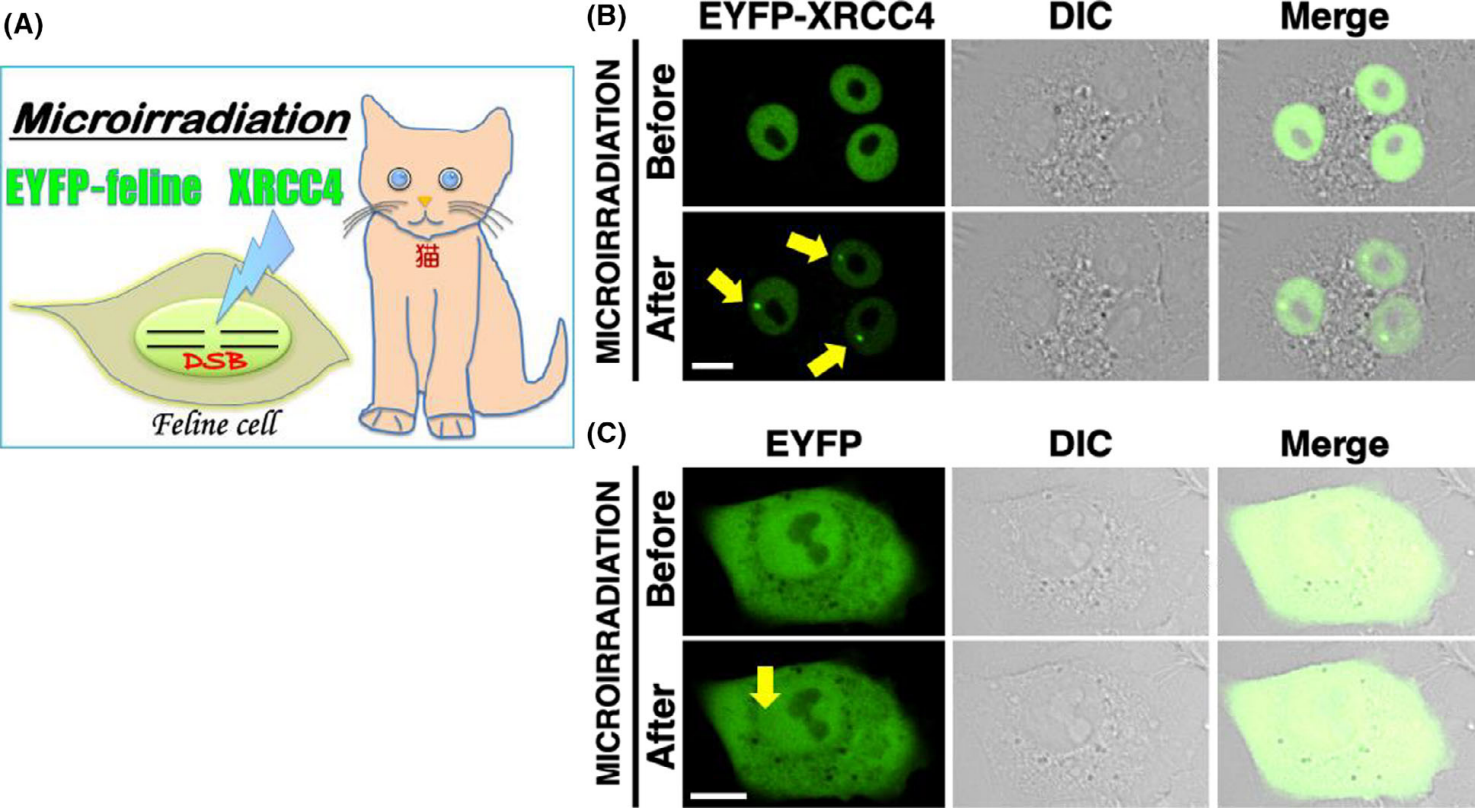
Accumulation of EYFP‐feline XRCC4 to DSBs induced by 405‐nm laser irradiation in CRFK cells. (A) The recruitment of EYFP‐feline XRCC4 to DSBs induced by 405‐nm laser irradiation in CRFK cells. Imaging of live CRFK cells transfected with pEYFP‐feline *XRCC4* (B) or pEYFP (control) (C) before (upper panel) and after (bottom panel) microirradiation. EYFP images for the same cells are shown alone (left panel) or merged (right panel) with the corresponding DIC (center panel) images. Arrows indicate the microirradiated site. Bar, 10 μm.

**Fig. 6:**
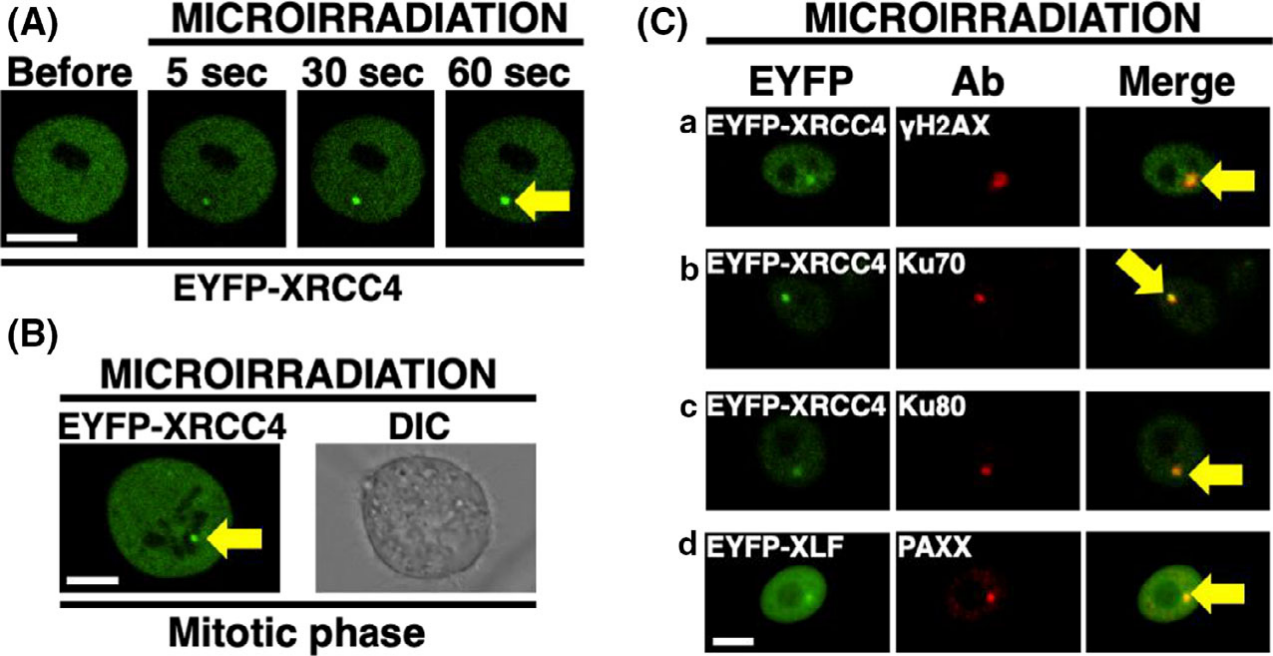
Rapid EYFP‐feline XRCC4 accumulation at DSBs with other NHEJ factors in CRFK cells. (A) Time‐dependent EYFP‐feline XRCC4 accumulation in live CRFK cells, from 5 to 60 s after irradiation. (B) EYFP‐feline XRCC4 accumulation at the microirradiated site in the mitotic cells. (C) Immunostaining of microirradiated cells transfected with pEYFP‐feline *XRCC4* or pEYFP‐feline *XLF* using anti‐γH2AX, anti‐Ku70, anti‐Ku80, or anti‐PAXX antibodies. Cells were fixed and stained with each antibody 5 min postirradiation. Left panel, EYFP‐feline XRCC4 (a, b, c) or EYFP‐feline XLF (d); center panel, γH2AX (a), Ku70 (b), Ku80 (c), PAXX (d); right panel, merged images. Arrows indicate the microirradiated site. Bar, 10 μm.

**Fig. 7:**
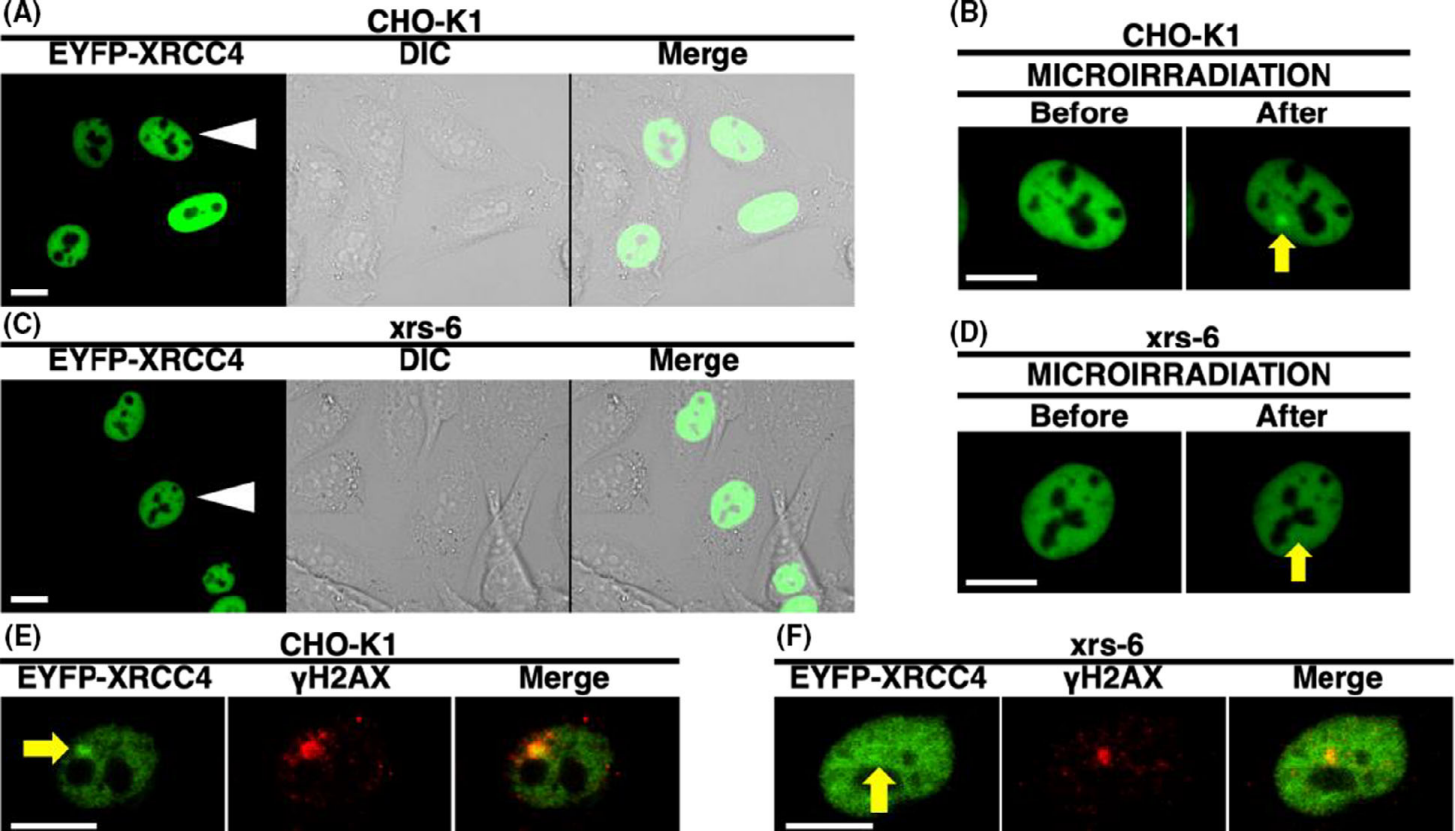
Ku‐dependent recruitment of EYFP‐feline XRCC4 to the sites of DSB induced by laser microirradiation. Subcellular localization of EYFP‐feline XRCC4 in the hamster ovary epithelial cell (CHO‐K1) (A, B) and the Ku80‐deficient CHO‐K1 mutant cell line (xrs‐6) (C, D). Feline XRCC4‐tagged with EYFP accumulated at irradiated sites in the CHO‐K1 (B), but not in the Ku80‐deficient xrs‐6 cells (D). Arrowheads and arrows indicate the microirradiated cell (A, C) and the microirradiated site (B, D), respectively. The microirradiated cells in (A) and (C) enlarged in (B) and (D), respectively. Each experiment was carried out on at least 10 cells and repeated three times. For the data presented, all the cells showed the same pattern. (E, F) Immunostaining of microirradiated cells transfected with pEYFP‐feline *XRCC4* using an anti‐γH2AX antibody. The CHO‐K1 (E) or xrs‐6 cells (F) were fixed and stained with the antibody 5 min after irradiation. Left panel, EYFP‐feline XRCC4; center panel, γH2AX; right panel, merged images. Arrows show the microirradiated sites. Bar, 10 μm.

It has been previously demonstrated that in humans, NHEJ proteins, including Ku70, Ku80, XLF, PAXX, and XRCC4, are recruited to the DSBs [12, 14, 39, 40] and that in feline cells, XLF accumulates and colocalizes with Ku70 and Ku80 at DSBs [31]. To investigate whether feline XRCC4 accumulates at the DSBs, DSBs were locally induced in EYFP‐feline XRCC4‐expressing CRFK cells or EYFP‐expressing cells using a 405‐nm laser (Fig. 5A). EYFP‐feline XRCC4—but not EYFP alone—accumulates at the microirradiated sites in live feline cells in the interphase (Fig. 5B,C). Time‐lapse imaging demonstrates that EYFP‐feline XRCC4 begins to accumulate in the microirradiated site within 5 s of irradiation (Fig. 6A). In addition, EYFP‐feline XRCC4 can accumulate at the microirradiated sites in feline cells in the interphase and during the mitotic phase (Figs 5B and 6A,B). Microirradiation in conjunction with immunostaining against γH2AX, Ku70, or Ku80 revealed that EYFP‐feline XRCC4 accumulates and colocalizes with Ku70 and Ku80 at DSBs in feline CRFK cells. Moreover, we observed that two other NHEJ factors, XLF and PAXX—which are members of the XRCC4 superfamily—accumulate and colocalize at DSBs in feline cells (Fig. 6C). Subsequently, we investigated whether Ku is necessary for feline XRCC4 accumulation. We first ascertained that EYFP‐feline XRCC4 is expressed and localized in the nuclei in both CHO‐K1 and xrs‐6 (Ku80‐deficient CHO‐K1 mutant) transfected with pEYFP‐feline *XRCC4* (Fig. 7A,C). Microirradiation in conjunction with immunostaining against γH2AX revealed that EYFP‐feline XRCC4 colocalized with γH2AX at the microirradiated sites in the CHO‐K1 cells, but not in the xrs‐6 cells, showing that the recruitment of EYFP‐feline XRCC4 depends on the presence of hamster Ku in the CHO‐K1 cells (Fig. 7B,D,E,F). In summary, these results suggest that Ku is critical for the feline XRCC4 recruitment to DSBs.

## Discussion

More than 60,000 cats are diagnosed with cancer annually in the United States [3]. In veterinary hospitals, resistance against chemotherapy and radiation therapy has become a serious problem (similar to human cancers). The NHEJ repair proteins including XRCC4 are considered promising target molecules for developing molecular‐targeted drugs to overcome resistance [3, 8, 9]. Our data revealed that the subcellular localization of feline XRCC4 is altered during the cell cycle and that XRCC4 is rapidly recruited to the DSBs in a Ku‐dependent manner. Comparative sequence analysis revealed that feline XRCC4 is more similar to human XRCC4 than to mouse XRCC4. Protein interaction domains and PTM sites required for the control of the spatiotemporal localization of XRCC4 were well conserved between humans and cats. Moreover, the amino acids in XRCC4 responsible for the development of MPD are perfectly conserved in cats. Our findings might be useful for understanding the functions of feline XRCC4 and the associated regulatory mechanisms, and to attain the basic information necessary for developing molecular‐targeted drugs against cancer and MPD.

In this study, we observed that feline XRCC4 is localized in the nuclei in the interphase and the cytoplasm in the mitotic phase, respectively. As described above, human XRCC4—that harbors the predicted NLS sequence—has been regarded as a nuclear protein—that functions only in the nuclei—since its discovery [12, 45]. The mechanisms mediated by the SUMOylation of XRCC4 at K210 have been proposed to regulate the nuclear translocation of XRCC4 in human cells [12, 46]. Previously, we had reported that the amino acid residue corresponding to the human XRCC4 K210, which is considered essential for nuclear localization, is not conserved in canine XRCC4 [35]. In this study, comparative analysis revealed that this amino acid residue is not conserved in feline XRCC4, whereas the putative NLS in human XRCC4 is highly conserved in feline XRCC4. Most recently, Maruoka et al. [47] reported that XRCC4 fragments function outside the nucleus. In response to apoptotic stimulation, XRCC4 is cleaved by caspases and the cleaved fragment is released into the cytoplasm, which in turn can directly regulate the function of the plasma membrane scramblase, Xkr4 during apoptosis. These results suggest that the caspase‐mediated change in the localization of the XRCC4 fragment is critical for XRCC4 function. Interestingly, our data showed that the caspase recognition sequence (_262_DVTD_265_) in human XRCC4 is perfectly conserved in feline XRCC4. We speculate that the localization of XRCC4 is regulated through its NLS and caspase recognition sequence, which are important for the spatiotemporal control of XRCC4 functions in cats and humans.

Our data showed that the sequences of feline XRCC4 are more similar to those of human XRCC4 than to mouse XRCC4. Our findings demonstrated that feline XRCC4 rapidly accumulates at DSBs in a Ku‐dependent manner. Additionally, our data showed that members of the XRCC4 superfamily, that is, XLF and PAXX accumulate at DSBs in feline cells, similar to the scenario in human cells [7, 14, 31], suggesting that feline cells may be a suitable model to investigate the mechanisms by which the activity of the XRCC4 superfamily proteins. Furthermore, the accumulation of feline XRCC4 was observed in interphase and mitotic phase cells. Terasawa et al. [48] reported that the phosphorylation of XRCC4 at S326 contributes to its M‐phase‐specific suppression of NHEJ repair. Phosphorylation of human XRCC4 and XLF results in the inhibition of the DNA bridging ability of XRCC4/XLF complexes at the DSBs [13]. CK2‐mediated phosphorylation at the threonine 233 residue of human XRCC4 mediates the interaction with an accessary NHEJ protein, APLF, and facilitates NHEJ [7]. Zhang et al. [43] demonstrated that FBXW7 facilitates NHEJ via K63‐linked polyubiquitylation at the K296 residue of human XRCC4. These phosphorylation and polyubiquitylation sites modified in human XRCC4 are conserved in feline XRCC4. Alternatively, miR‐129‐3p has been reported to inhibit the NHEJ pathway—via the inhibition of XRCC4 SUMOylation—by targeting the SUMO‐activating enzyme SAE1 and repressing the progression of human gastric cancer [49]. SAE1 suppression is thought to result in the inhibition of the nuclear localization of XRCC4 and lack of the activation of NHEJ. Human SUMO2 is deposited at the SUMO2‐binding sequence SIM‐1 in XRCC4 and regulates XRCC4 recruitment at the DNA damage sites [44]. In this study, we found that the SIM‐1 of feline XRCC4 is conserved. We speculate that the SUMOylation is required for the recruitment of feline XRCC4 at the DSBs and NHEJ activity regulation, although further studies are needed to confirm this. Interestingly, a missense mutation (W43R)—responsible for the development of MPD—was located close to the SIM‐1 and this mutation strongly reduced the binding of SUMO with XRCC4 [30, 44]. The other MPD‐causing mutation (R275*) is located close to the caspase recognition sequence, and the mutation appears to induce the formation of a cleaved fragment of XRCC4 (similar to caspase‐dependent XRCC4 fragments). Our comparative analysis showed that all target amino acids examined—including the Tryptophan residue at position 43 and the Arginine at position 275—which are targeted for the MPD mutation are completely conserved in feline XRCC4. Therefore, studies on feline XRCC4 can be useful for investigating the regulation of XRCC4 by various PTDs and protein interactions, and for model studies of human diseases.

Various mutations in the NHEJ repair genes are known to induce the development of hereditary disease with radiosensitivity and/or immunodeficiency in animals and humans. Mutations in *DNA‐PKcs* are responsible for inducing radiosensitive severe combined immunodeficiency in humans, dogs, mice, and horses [50, 51]. Human XLF or DNA Ligase IV mutations are known to induce the development of XLF syndrome or Ligase IV syndrome, respectively, which are genetic diseases resulting in immunodeficiency and neurological disorders, respectively [52, 53]. XLF plays an essential role in DSB repair in the brain and neural tube of chick embryos, similar to the scenario observed in humans, but not in mice [18, 54]. As described above, certain XRCC4 mutations induce the development of MPD in humans [30]. Thus, further studies pertaining to feline NHEJ repair genes may lead to the discovery of new diseases in cats caused by XRCC4 and other NHEJ gene mutations.

Radio‐resistance in cats and feline tumor radiation therapy resistance are well recognized in veterinary hospitals [5]. However, not many studies have investigated feline NHEJ repair, which is likely to be the cause of these resistances, and the mechanisms at work remain unclear. By linking basic research in veterinary and human medicine, the development of treatments for humans, cats, and dogs should be accelerated. Our findings suggest that comparative studies on NHEJ repair using feline and human cells may serve as a good model for elucidating the regulatory mechanisms of XRCC4 functions and NHEJ repair. This and further studies might be useful to elucidate the role of NHEJ in feline radio‐resistance and the role of abnormal XRCC4 in diseases, including cancers and MPD, and to develop XRCC4‐targeted drugs, such as radiosensitizer, for humans and cats.

## Conflict of interest

The authors declare no conflict of interest.

## Author contributions

MK and AK designed and directed the experiments. MK, YY, and AK carried out experiments and the analysis of the results. MK wrote the manuscript, and YY and AK read and approved the final paper.

## Data Availability

The sequence of feline *XRCC4* cloned in this study has been deposited to the DDBJ/EMBL/NCBI database [accession number: LC309245].
